# Central line-associated bloodstream infection rates in intensive care units of China’s hospitals: a meta-analysis

**DOI:** 10.3389/fpubh.2025.1480428

**Published:** 2025-04-16

**Authors:** Minghong Cai, Xiaoyan Jiang, Jing Chen, Jiayu Wu, Yu Lyu, Qian Xiang

**Affiliations:** Healthcare-Associated Infection Control Center, Sichuan Academy of Medical Sciences, Sichuan Provincial People's Hospital, School of Medicine, University of Electronic Science and Technology of China, Chengdu, Sichuan, China

**Keywords:** central line-associated bloodstream infection, ICU, China, meta-analysis, CLABSI

## Abstract

**Background and aim:**

Various reasons have hindered accurate reporting of the central line-associated bloodstream infection (CLABSI) rates in China’s hospital ICUs. This study conducts a meta-analysis to provide a precise assessment of CLABSI rates in these units.

**Methods:**

Adhering to the PRISMA guidelines, a systematic search was conducted in PubMed, Web of Science, Embase, CNKI, Wanfang and Weipu from January 2008 to December 2023. Selection of literature followed strict criteria, ensuring relevant data extraction. A random-effects model facilitated the meta-analysis.

**Results:**

The analysis incorporated 23 studies (PubMed: 5, Web of Science: 4, Embase: 1, CNKI: 9, Wanfang: 2, Weipu: 2). It revealed an overall weighted CLABSI rate in China’s ICUs of 2.65‰ (95% CI: 2.57–2.73‰), with 3.12‰ in adult ICU (95% CI: 2.70–3.54‰) and 2.57‰ in pediatric ICUs (95% CI: 2.49–2.66‰). Notably, adult ICUs in North of China recorded the highest rate at 5.13‰ (95% CI: 4.23–6.02‰), and pediatric ICUs in East of China had 3.35‰ (95% CI: 2.85–3.85‰).

**Conclusion:**

The study indicates that CLABSI rates in China’s ICUs surpass those reported in national healthcare reports and the US CDC-NHSN data. This underscores the urgency for enhanced surveillance and infection control. The findings stress the need for standardized surveillance definitions and methods to truly represent CLABSI epidemiology and develop effective prevention strategies.

## Introduction

Central line-associated bloodstream infection (CLABSI) is one of the common healthcare-associated infections (HAIs) encountered in intensive care units (ICUs), adversely affecting patient prognoses and resulting in significant economic costs. Surveillance of HAIs initiated by the International Nosocomial Infection Control Consortium (INICC) across various countries globally indicates that the CLABSI rates range from 2–147.3 per 1,000 central line days. CLABSI is associated with an additional mortality rate of 3.2 to 75.1%, an increase of 1.3–26.2 days in hospital stay, and additional costs ranging from US $ 4,888.42 to11,590.93 (1).

In February 2021, the National Health Commission of China established the 2021 National Medical Quality and Safety Improvement Goals, which included the reducing the incidence rate of intravascular catheter-related bloodstream infections as the ninth goal. It emphasized the need for comprehensive measures to intervene, ensuring medical safety and patient rights. The National Medical Service and Quality Safety Report reveals considerable variation in the reported CLABSI rates across different medical specialties. The specialty of intensive care medicine has reported a rate of 2.07–3.10 per 1,000 central line days (2), whereas the specialty of HAI management reported a notably lower rate of only 0.57–1.01per 1,000 central line days (3). While these discrepancies may be due to differences in the hospitals and time frames of the reports, other factors cannot be overlooked, such as unclear definitions for surveillance, subjective methods of surveillance, and varying definitions of the same indicators among different specialties. The actual CLABSI rates in ICUs of Chinese hospitals remains unclear and may be underestimated.

To address these challenges and provide a more accurate depiction of CLABSI rates in ICUs of Chinese hospitals, our study conducted a comprehensive meta-analysis. Relevant data from both Chinese and English literature rigorously selected and published from January 2008 to December 2023 were collated.

## Methods

### Search strategy

Following the guidelines of the Preferred Reporting Items for Systematic Reviews and Meta-Analyses: PRISMA Statement, a comprehensive literature search was conducted in the PubMed, Web of Science, Embase, CNKI, Wanfang, and Weipu database. For each database, the search strategy was provided in the Supplementary file. All the literature published from January 2008 to November 2023 were searched.

### Selection criteria

#### Inclusion criteria

Descriptive studies about or involving CLABSI in Chinese hospitals ICUs.

#### Exclusion criteria

Abstract Exclusion Criteria: (1) non-descriptive (experimental) studies and analytical studies such as case–control studies and cohort studies. (2) Studies not involving CLABSI. (3) Studies with subjects who are not patients in ICUs.

Full-text Exclusion Criteria: (1) studies solely focusing on CLABSI caused by a specific pathogen. (2) Catheters other than central lines, such as arterial catheters, arteriovenous fistulas, atrial catheters, extracorporeal membrane oxygenation (ECMO), hemodialysis catheters, intra-aortic balloon pump (IABP) devices, peripheral veins catheters, ventricular assist devices (VAD), etc. (3) Studies that do not describe the definition of CLABSI, or use a definition of CLABSI that is inconsistent with the definition by the U.S. CDC NHSN, or only describe the use of China’s Nosocomial Infection Diagnostic Criteria (2001 Trial version) as definition. (4) Studies with original data that only report the CLABSI rates without reporting the number of central line days or the number of CLABSI cases.

### Data extraction

The following information from the eligible full texts was extracted: title, the first author, publication year, study period, city or region, number of hospitals, number of ICUs, catheter type, number of CLABSI cases, and central line days. Data verification was performed through cross-checking by two researchers.

### Quality assessment

Two researchers independently conducted literature screening and quality assessment based on inclusion and exclusion criteria. In cases of disagreement, a third researcher was involved in the discussion to resolve the issue. The Strengthening the Reporting of Observational Studies in Epidemiology (STROBE) statement checklist was used to assess the quality of all studies. The standards were as follows: High Quality (meeting more than 75% of the criteria), Medium Quality (meeting 50–75% of the criteria), and Low Quality (meeting less than 50% of the criteria).

### Statistical analysis

All statistical analyses were performed with Statistical Software-STATA, version 18.0. A meta-analysis was conducted using the Metaprop command. The CLABSI rate = the number of CLABSI cases/the number of central line days*1000. The pooled and weighted CLABSI rates, along with the 95% Confidence Intervals (95% CI), were calculated within a random-effects model and represented in forest plots. Heterogeneity was evaluated using the *I*^2^ index, and given the substantial heterogeneity (*I*^2^ > 75%), we employed a random-effects model, which is methodologically more appropriate for meta-analysis with considerable inter-study variability. The subgroup analysis was subjected based on ICU types and regions. During the subgroup analysis, different types of adult ICUs were categorized into medical ICUs, surgical ICUs, and general ICUs according to their specialty. When the city of the ICU was not clearly identifiable, the subgroup analysis was conducted based on the region. The subgroup analysis was performed to characterize the distribution of CLABSI rates across different ICU types and geographic regions, rather than to test for intergroup differences. Due to the imbalanced sample sizes among subgroups, formal statistical comparisons between groups were not conducted. Publication bias was assessed by funnel plots.

## Results

### Search results

**Figure 1:**
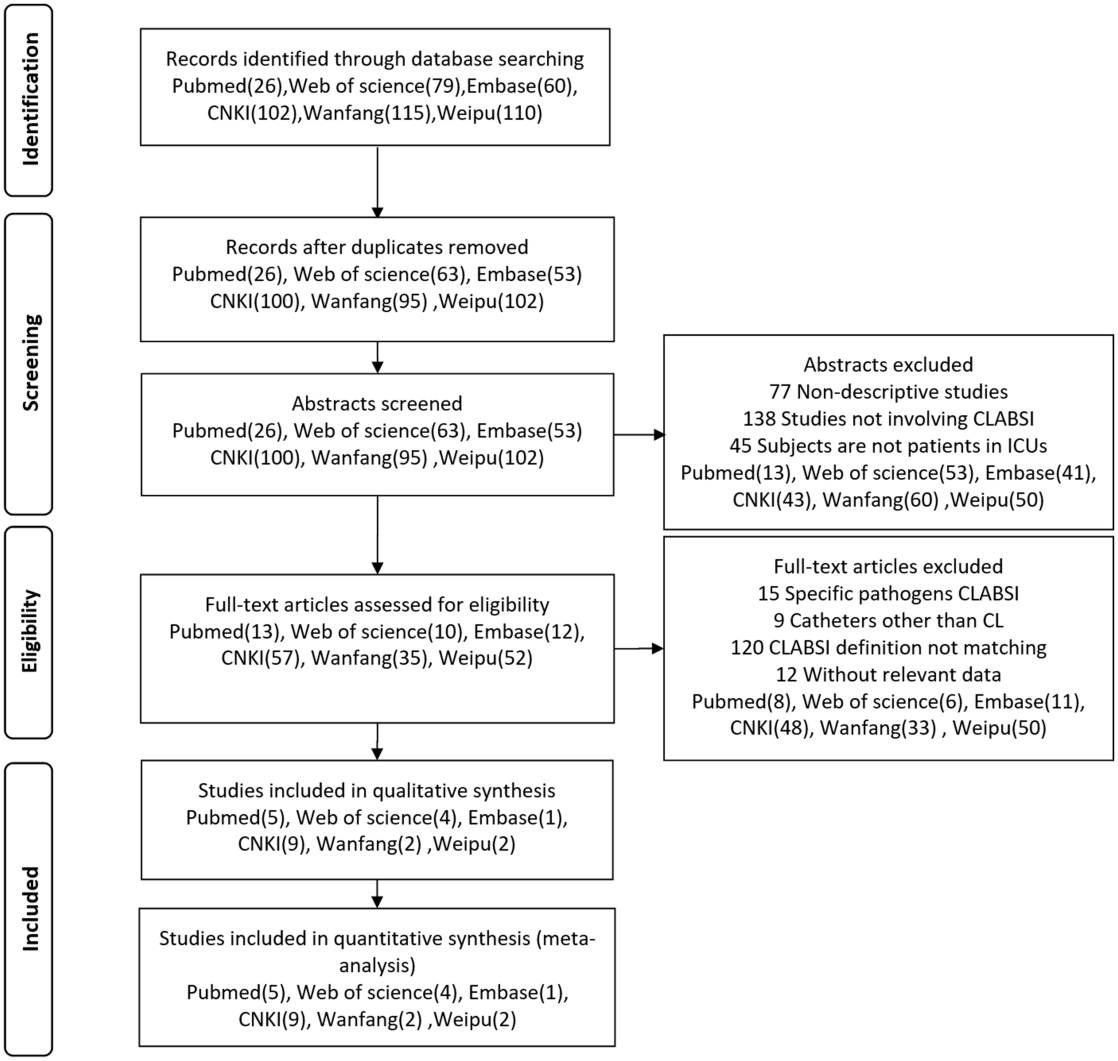
Screening and selection process of studies.

We retrieved 395 relevant articles from the PubMed (26), Web of Science (79), Embase (60), CNKI (102), Wanfang (115) and Weipu (110) databases, which were reduced to 333 after removing duplicates. Upon initial screening of titles and abstracts, 260 articles were excluded, and another 156 were excluded after full-text review. Ultimately, 23 articles (PubMed: 5, Web of Science: 4, Embase: 1, CNKI: 9, Wanfang: 2, Weipu: 2) were included for data extraction and analysis. Figure 1 showed the literature screening and selection process.

### Studies included for analysis, quality assessment and publication bias

**Figure 2:**
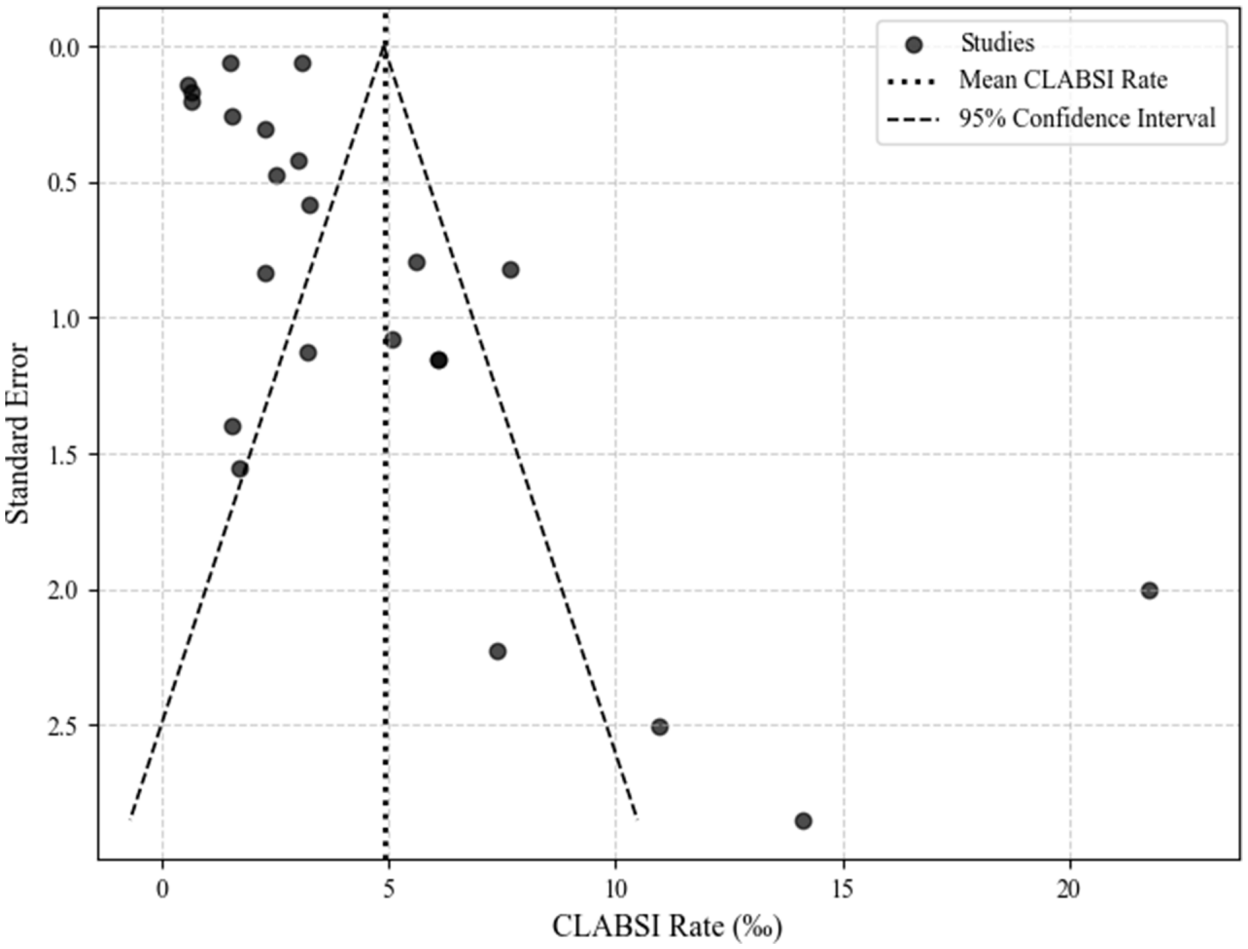
The funnel plot of CLABSl rates of included studies.

This manuscript encompasses an analysis of 23 studies, spanning from 2008 to 2023, which report on the CLABSI rates in various types of ICUs across different cities and areas. The quality of these studies was rigorously assessed using the STROBE checklist. Information and quality assessment results of each study are presented in Table 1. The funnel plot revealed that the studies included in this analysis were distributed asymmetrically outside the funnel’s symmetry range, suggesting the presence of publication bias. The publication bias assessment funnel plot is detailed in Figure 2.

**Table 1:** Overview of studies included in the analysis and quality assessment results.

Author	City/province	Study Period	ICU type	Number of hospitals	Number of ICUs	Type of catheter	CL-days	Number of CLABSI Cases	CLABSI rate (‰ 95%CI)	Quality level
Hei et al. (8)	Changsha	Mar 2008 – Feb 2011	Neonatal ICU	1	1	UVC	1909	27	14.14 (9.38–20.55)	High
Peng and Lu et al. (9)	Shenyang	Jun 2007 – May 2008	General ICU	1	1	CVC	1913	21	10.98 (6.82–16.64)	High
Tao et al. (10)	Shanghai	Sep 2004 – Dec 2009	Cardiac Surgery ICU, Coronary ICU, General ICU, Medical ICU, Neurosurgery ICU, Pediatric ICU, Respiratory ICU, Surgical ICU, Trauma ICU	70	398	CL	835,315	2,578	3.09 (2.97–3.21)	High
Hu et al. (11)	Shanghai, Beijing, Xi’an, Taiyuan	Aug 2008 – Jul 2010	Cardiology ICU, General ICU, Neurology ICU, Cardiology ICU, Surgical ICU	4	7	CL	12,410	95	7.66 (6.18–9.41)	High
Wang et al. (12)	Xi’an	2013–2015	Respiratory ICU	1	1	CL	1,622	12	7.40 (3.89–12.61)	High
Ren et al. (13)	Beijing, Shanxi, Shandong, Jiangsu, Zhejiang, Henan, Hunan, Chongqing, Guangdong	Oct 2013 – Sep 2014	Neonatal ICU	17	17	CL (UVC and PICC)	19,621	13	0.66 (0.35–1.16)	High
Wang et al. (14)	Yinchuan	Jan 2013 – Dec 2022	General ICU	1	1	CL	36,507	22	0.60 (0.38–0.93)	Medium
Wang et al. (15)	Hefei	2017–2019	General ICU	1	1	CL	5,243	32	6.11 (4.18–8.71)	High
Liu et al. (16)	Shanghai	2015–2017	General ICU	5	5	CL	26,609	18	0.68 (0.41–1.07)	Medium
Jia et al. (17)	Beijing	2011–2016	Respiratory ICU	1	1	CL	12,182	31	2.54 (1.75–3.61)	High
Zhang et al. (18)	Xuzhou	2015	General ICU	23	23	CL	24,286	38	1.56 (1.12–2.14)	High
Zha and Zhang (19)	Shanghai	2011	Coronary ICU, Emergency ICU, Respiratory ICU, Surgical ICU, Cardiac Surgery ICU	1	6	CL	10,103	33	3.27 (2.27–4.56)	High
Mao et al. (20)	Xuzhou	Jun 2010 – Dec 2011	General ICU	1	1	CL	5,070	31	6.11 (4.20–8.73)	High
Li et al. (21)	Chengdu	Jan 2014 – Dec 2014	Respiratory ICU	1	1	CL	1,270	2	1.57 (0.19–5.67)	Medium
Fan et al. (22)	Haikou	2018	General ICU	1	1	CL	3,966	9	2.27 (1.04–4.31)	Medium
Deng et al. (23)	Chengdu	Oct 2014-Sep 2015	General ICU	1	1	CL	1,147	2	1.74 (0.21–6.30)	Medium
Wang et al. (24)	Dongguan	Jan 2015–Nov 2015	General ICU	1	1	CL	2,485	8	3.22 (1.39–5.81)	Medium
Guan et al. (25)	Shanghai	Jan 2008–Jul 2009	General ICU	1	1	CL	4,339	22	5.07 (2.95–7.19)	Medium
Li et al. (26)	Hefei	2012–2019	General ICU	1	1	CL	23,830	54	2.27 (1.66–2.87)	Medium
Xu et al. (27)	Hangzhou	Jun 2018–May 2020	Neonatal ICU	1	1	PICC	8,929	50	5.60 (4.05–7.15)	Medium
Zeng et al. (28)	11 provinces/municipality	Jan 2014–Dec 2018	General ICU, Emergency ICU, Surgical ICU, Neurological ICU, Respiratory ICU, Medical ICU, Coronary ICU, Pediatric ICU	62	79	CVC	466,585	702	1.50 (1.39–1.62)	High
Zheng et al. (29)	24 provinces	Nov 2019–Aug 2021	Neonatal ICU	44	44	UVC	17,168	52	3.03 (2.21–3.85)	High
Wang et al. (30)	Chongqing	Jan 2018–Dec 2021	Burn ICU	1	1	CVC	5,431	118	21.73 (17.81–25.65)	Medium

UVC, umbilical venous catheter; CVC, central venous catheter; PICC, peripherally inserted central catheter; CL, central line.

### The CLABSI rates in different types of ICUs

The overall weighted CLABSI rate across all ICU types is 2.65‰ (95% CI: 2.57–2.73‰). A notable observation is that pediatric ICUs exhibit a slightly higher rate compared to adult ICUs, with pediatric ICUs at 3.12‰ (95% CI: 2.70–3.54‰) versus 2.57‰ (95% CI: 2.49–2.66‰) in adult ICUs. Within the adult ICU category, Surgical ICUs recorded the highest rate at 2.92‰ (95% CI, 2.78–3.07‰). Table 2 provides a detailed breakdown of pooled and weighted CLABSI rates across different ICU types.

**Table 2:** The pooled and weighted CLABSI rates in different types of ICUs.

ICU type	Number of ICUs	Number of CLABSI cases	CL-days	Pooled CLABSI rate (‰, 95%CI)	Weighted CLABSI rate (‰, 95%CI)
Adult ICU	510	3,760	1,460,606	2.57 (2.49–2.66)	2.57 (2.49–2.66)
Medical ICU	183	569	197,530	2.88 (2.65–3.13)	2.88 (2.64–3.12)
Respiratory ICU	56	145	59,975	2.42 (2.06–2.84)	2.42 (2.02–2.81)
Medical ICU	57	173	64,449	2.68 (2.31–3.11)	2.68 (2.28–3.08)
Neurology ICU	6	10	8,262	1.21 (0.66–2.23)	1.21 (0.46–1.96)
Coronary ICU	64	241	64,844	3.72 (3.28–4.22)	3.72 (3.25–4.18)
Surgical ICU	182	1,518	519,324	2.92 (2.78–3.07)	2.92 (2.78–3.07)
Trauma ICU	9	6	5,394	1.11 (0.51–2.42)	1.11 (0.22–2.00)
Neurosurgery ICU	43	145	64,521	2.25 (1.91–2.64)	2.25 (1.88–2.61)
Surgical ICU	72	912	272,114	3.35 (3.14–3.58)	3.35 (3.13–3.57)
Cardiac Surgery ICU	49	337	169,896	1.98 (1.78–2.21)	1.98 (1.77–2.20)
Burn ICU	9	118	5,782	20.41 (17.07–24.38)	20.41 (16.76–24.05)
General ICU	144	1,673	743,752	2.25 (2.14–2.36)	2.25 (2.14–2.36)
Emergency ICU	7	23	13,001	1.77 (1.18–2.65)	1.77 (1.05–2.49)
General ICU	137	1,650	730,751	2.26 (2.15–2.37)	2.26 (2.15–2.37)
Pediatric ICU	84	210	67,321	3.12 (2.73–3.57)	3.12 (2.70–3.54)
Pediatric ICU	21	68	19,694	3.45 (2.72–4.37)	3.45 (2.63–4.27)
Neonatal ICU	63	142	47,627	2.98 (2.53–3.51)	2.98 (2.49–3.47)
Overall	593	3,970	1,527,927	2.60 (2.52–2.68)	2.65 (2.57–2.73)

### The CLABSI rates in adult and pediatric ICUs

**Figure 3:**
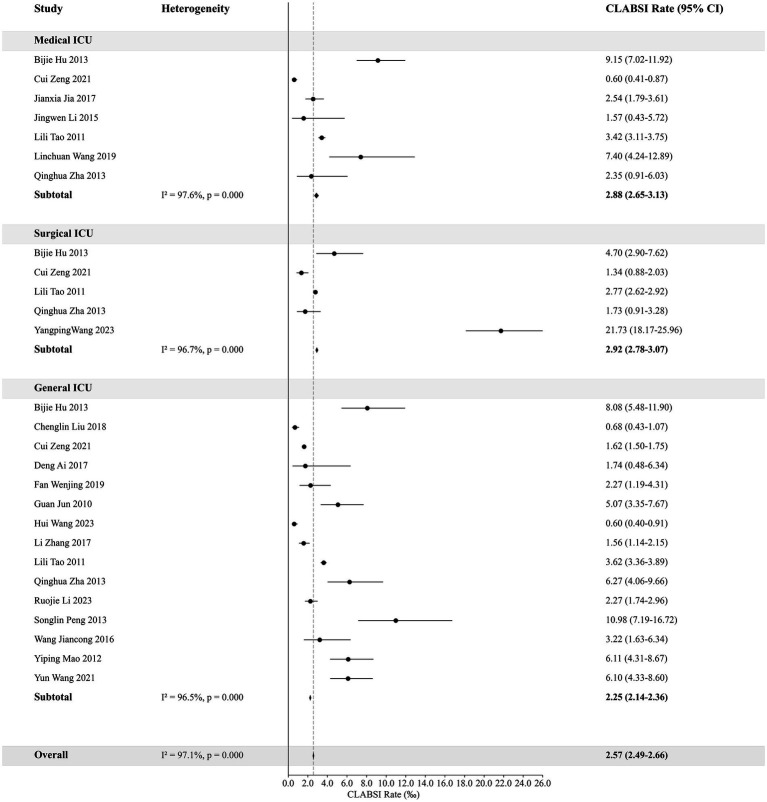
The weighted rates of CLABSI in adult ICUs.

**Figure 4:**
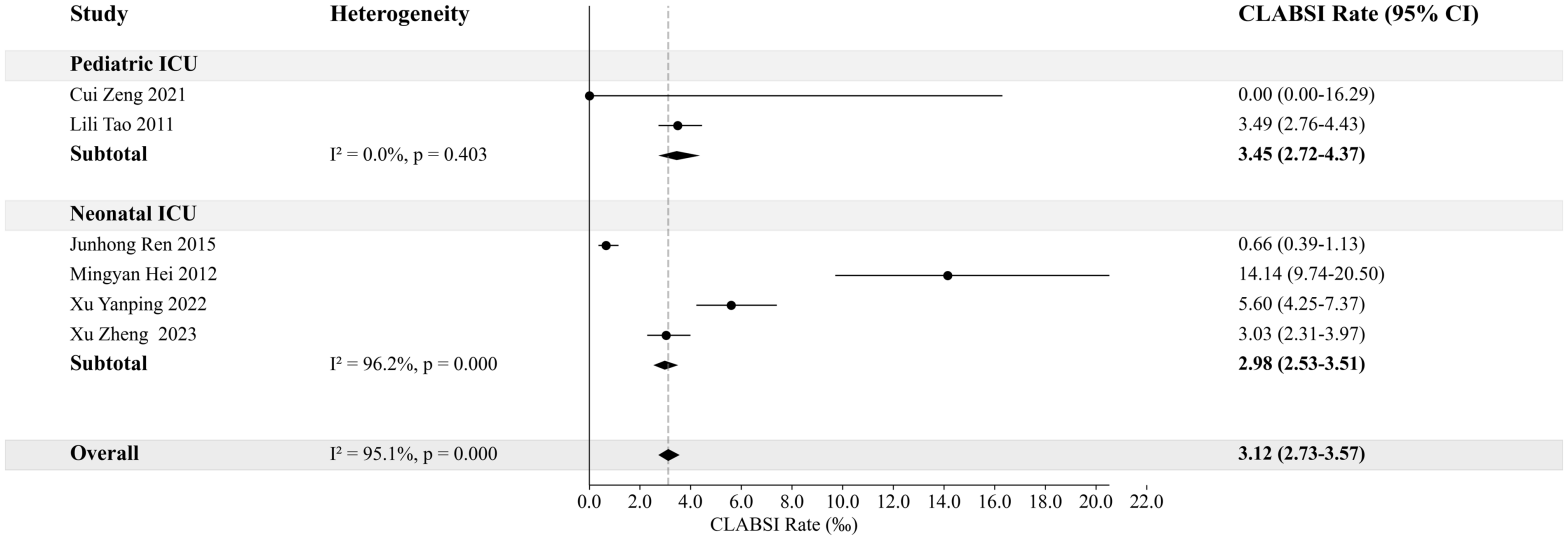
The weighted rates of CLABSI in pediatric ICUs.

This analysis categorizes adult ICUs based on their specialty into Medical ICU, Surgical ICU, and General ICU, and compares their CLABSI rates. The weighted rate for Medical ICU is 2.88‰ (95% CI: 2.65–3.13‰), for Surgical ICU is 2.92‰ (95% CI: 2.78–3.07‰), and for General ICU is 2.25‰ (95% CI: 2.14–2.36‰). For pediatric ICUs, the weighted rates are 3.45‰ (95% CI: 2.72–4.37‰) for Pediatric ICU and 2.98‰ (95% CI: 2.53–3.51‰) for Neonatal ICU. Figures 3, 4 provide detailed visual representations of these data.

### The CLABSI rates in ICUs of different regions

**Figure 5:**
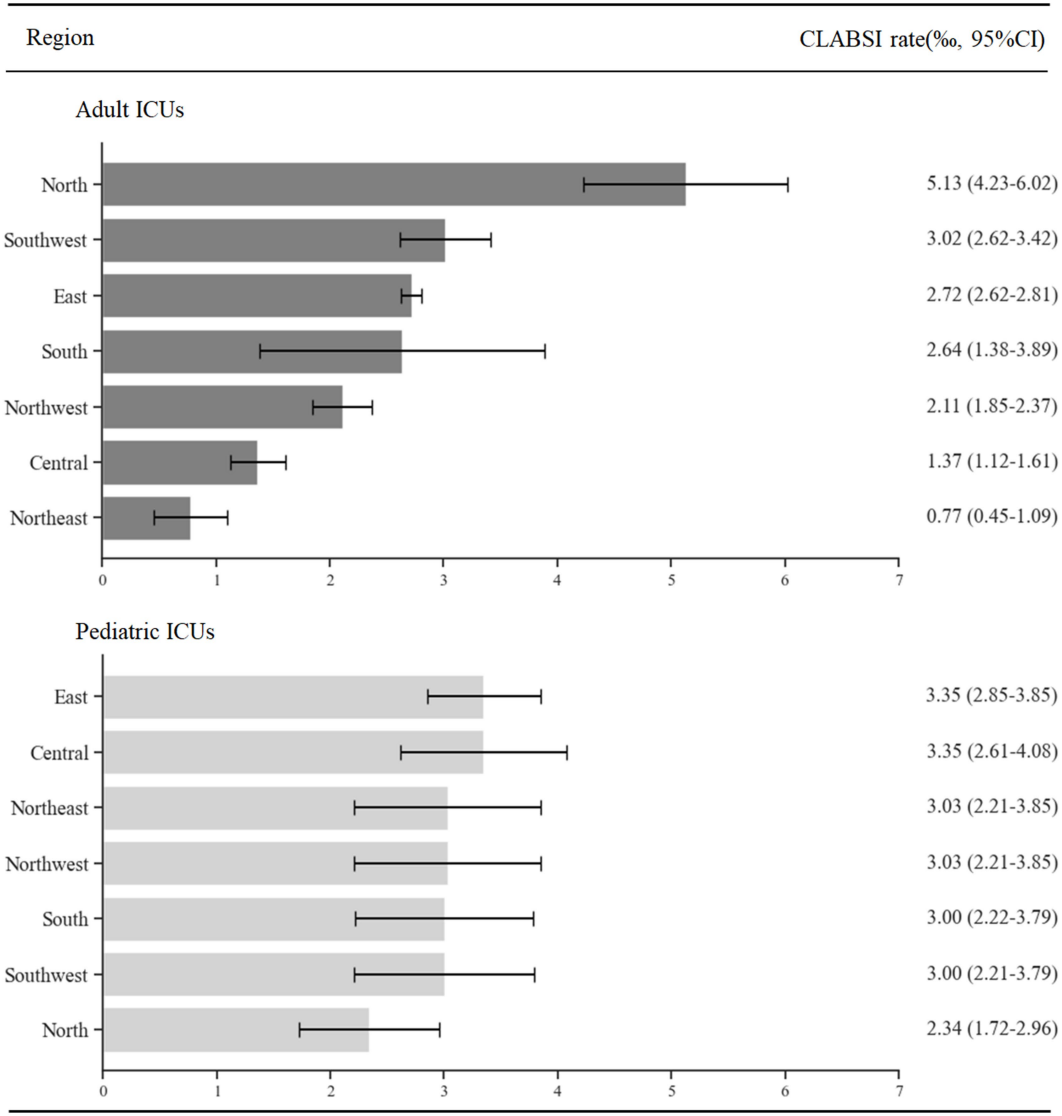
The weighted rates of CLABSI in ICUs of regions.

This section presents a detailed analysis of CLABSI rates in ICUs, organized by the region. The data, derived from various studies, categorizes hospitals by their location. For adult ICUs, the CLABSI rates, in descending order, are observed as follows: North, Southwest, East, South, Northwest, Central and Northeast of China, in pediatric ICUs, the rates follow this sequence from highest to lowest: East, Central, Northeast, Northwest, South, Southwest and North of China. Figure 5 provides a comprehensive display of the weighted rates of CLABSI in the ICUs across each mentioned region.

## Discussion

In the literature on catheter-associated bloodstream infections, two definitions are commonly discussed: Catheter-Related Bloodstream Infection (CRBSI) and Central Line–Associated Bloodstream Infection (CLABSI). CRBSI, a clinical definition used for diagnosis, involves the patient’s symptoms and requires the culture of the same pathogen from both peripheral and catheter blood, often showing higher bacterial quantification in the latter. This definition pinpoints the catheter as the bacteria source (4). Despite its rigor, CRBSI’s practical application in clinical settings faces challenges, such as the lack of quantitative cultures in some hospitals, suboptimal practices in blood culture sample submission, and a generally low positivity rate in blood cultures. These factors could lead to an underestimation of CRBSI incidence. On the other hand, CLABSI is an epidemiological definition used for disease surveillance, focusing on laboratory-confirmed bloodstream infections in patients with central lines, when no other infection source is identified (5). The CDC in the United States has adopted CLABSI for surveillance in NHSN reports since 2008 due to its greater sensitivity and accessibility compared to CRBSI, making it a globally used definition for HAI surveillance and infection control quality improvement. This is the reason why January 2008 was selected as the starting point for the literature search in this study.

The National Health Commission of China, recognizing these international standards, integrated both CRBSI and CLABSI definitions in its 2010 Guideline for Prevention and Control of Catheter-Related Bloodstream Infection (Trial version). This guideline, however, did not mandate simultaneous positivity and identical pathogens in peripheral and catheter blood, leaning closer to the CLABSI definition (6). Further expanding the scope, the 2021 Guideline for Prevention and Control of Vessel Catheter-Associated Infection included all intravascular catheters and both bloodstream and local infections at the insertion site (7). Despite these advancements, discrepancies in catheter duration definitions among CRBSI, CLABSI, and VCAI persist (4–7), with clinicians often favoring CRBSI, potentially leading to underreported incidence rates. Our study, focusing on clarity and precision, favored studies using CLABSI or similar definitions, aiming to reduce the impact of these discrepancies.

Our study revealed that the CLABSI rates in Chinese hospital ICUs (both adult and pediatric) were higher than the rates reported by the NHSN for American hospital ICUs (adult ICU: 2.57‰ vs. 0.93–1.71‰, pediatric ICU: 3.12‰ vs. 1.22–2.23%, neonatal ICU: 2.98‰ vs. 1.13–2.28‰). However, these rates were lower than those reported by the INICC (showed in Table 3), reflecting the National Health Commission of China’s and hospitals’ achievements in HAI prevention and control. The 2010 guideline played a pivotal role in reducing CLABSI rates in China by raising healthcare personnel awareness and compliance with prevention and control practices. Yet, this also underlined the existing gaps in CLABSI prevention and control between Chinese and U.S. hospitals. China needs to continue striving for improvements in policy enforcement, healthcare personnel training, medical resource optimization, and HAI surveillance enhancement.

**Table 3:** CLABSI rates of different type ICUs reported by NHSN and INICC.

	Adult ICU (‰, 95% CI)	Pediatric ICU (‰, 95% CI)	Neonatal ICU (‰, 95% CI)
NHSN			
2009 (31)	1.71(1.63–1.78)	2.23(2.05–2.41)	2.28(2.15–2.41)
2010 (32)	0.94(0.91–0.97)	1.82(1.69–1.95)	1.63(1.53–1.73)
2011 (33)	0.68(0.27–1.32)	1.72(0.89–3.15)	1.55(1.46–1.63)
2012 (34)	0.96 (0.92–1.00)	1.41(1.35–1.47)	1.30 (1.27–1.33)
2013 (35)	0.93(0.89–0.97)	1.22(1.11–1.33)	1.13(1.10–1.15)
INICC			
2007–2012 (36)	4.67(4.58–4.76)	6.07(5.66–6.49)	5.17(4.92–5.42)
2010–2015 (37)	4.42(4.36–4.48)	8.47(7.76–9.18)	16.37(15.22–17.64)
2012–2017 (38)	4.85(4.80–4.90)	7.19(6.54–7.84)	12.73(11.74–13.72)
2013–2018 (39)	4.61(4.48–4.74)	11.21(11.69–11.75)	–

NHSN, National Healthcare Safety Network of the Centers for Disease Control and Prevention (CDC). INICC, International Nosocomial Infection Control Consortium.

However, significant challenges remain in standardizing China’s HAI surveillance. The overall CLABSI rate showed in our study was slightly higher than that reported in the National Medical Service and Quality Safety Report for the intensive care specialty (2.65‰ vs. 0.57–3.1‰), and significantly higher than the data reported by the HAI management specialty (2.65‰ vs. 0.57–1.01‰). Discrepancies in CLABSI rates between different data sources highlight these challenges, emphasizing the need for updated HAI definitions and surveillance method standardization.

Our study also had limitations, such as strict inclusion and exclusion criteria preventing comprehensive data aggregation from numerous Chinese hospitals. Particularly, the number of studies meeting the criteria for CLABSI in pediatric and neonatal ICUs is extremely limited. Nonetheless, this stringent approach preserved the integrity of our meta-analysis results. Secondly, there was a high degree of heterogeneity among the studies, although methods such as employing a random-effects model and conducting subgroup analyses were attempted. This might be related to the differences in time periods, geographical regions, hospital levels, healthcare service quality, and adherence to infection prevention and control measures, particularly those specific to CLABSI prevention in the included studies. Lastly, since the data were not from a systematic surveillance system, it was not feasible to categorize and analyze the data by year, nor to demonstrate the dynamic trends in CLABSI rates. We believe the ongoing development of the Chinese Medical Institution Infection Surveillance System (CMIISS) will address these issues.

## Data Availability

The original contributions presented in the study are included in the article/Supplementary material, further inquiries can be directed to the corresponding authors.
