# Supplementary material for: Central line-associated bloodstream infection rates in intensive care units of China’s hospitals: a meta-analysis
Source: Front Public Health. 2025 Apr 16;13:1480428. doi: 10.3389/fpubh.2025.1480428 (PMC12040942; doi:10.3389/fpubh.2025.1480428)
Supplement: Supplementary file 1 [file Table_1.docx]

**The Search Strategy of Databases**

PubMed database : ‘Catheter Related Infections’ (MeSH term) or ‘CLABSI’ or ‘Device Associated Infection’ and (‘Incidence’ (MeSH term) or ‘Epidemiology’ (MeSH term) or ‘Epidemiological monitoring’ or ‘Surveillance’)and ‘China’ (MeSH term).

Web of science database: ((TS=("Catheter Related Infection*" OR "Device Associated Infection*" OR "central line-associated bloodstream infection*" OR "CLABSI" OR "Central Venous Catheter Infection*" OR "Catheter-Associated Bloodstream Infection*") OR TI=("Catheter Related Infection*" OR "Device Associated Infection*" OR "central line-associated bloodstream infection*" OR "CLABSI" OR "Central Venous Catheter Infection*" OR "Catheter-Associated Bloodstream Infection*")) AND ( TS=("Intensive Care Unit*" OR "Critical Care Medicine" OR "ICU" OR "Critical Care Unit*" OR "CCU") OR TI=("Intensive Care Unit*" OR "ICU")) AND (TS=("China" OR "Chinese") OR AD=China))

Embase database: ( ( 'catheter related infection'/exp OR 'device associated infection'/exp OR 'central line-associated bloodstream infection'/exp OR 'clabsi'/exp OR ( (ti,ab,kw=("catheter-related infection*" OR "device-associated infection*" OR "central line-associated bloodstream infection*" OR "CLABSI" OR "central venous catheter infection*" OR "catheter-associated bloodstream infection*")))) AND ( 'intensive care unit'/exp OR 'critical care'/exp OR (ti,ab,kw=("intensive care unit*" OR "critical care medicine" OR "ICU" OR "critical care unit*" OR "CCU"))) AND ( country=china OR (ad=china) OR (ti,ab,kw=("China" OR "Chinese"))))

CNKI database: (KY='Intensive Care Unit' OR TI='Intensive Care Unit' OR KY='ICU Ward' OR TI='ICU Ward' OR KY='Critical Care Medicine' OR TI='Critical Care Medicine' OR KY='ICU' OR TI='ICU') and (KY='Target Monitoring' or KY='Catheter Related Bloodstream Infections' or KY='Device Associated Infections' OR KY='Central Venous Catheter Bloodstream Infections' OR KY='Catheter-Associated Infections').

Wanfang database:( (SU=("catheter-related infection" OR "catheter-associated bloodstream infection" OR "device-associated infection" OR "central venous catheter bloodstream infection" OR "CLABSI") OR TI=("catheter-related infection" OR "catheter-associated bloodstream infection" OR "device-associated infection" OR "central venous catheter bloodstream infection" OR "CLABSI")) AND (SU=("infection surveillance" OR "targeted surveillance" OR "hospital-acquired infection surveillance") OR TI=("infection surveillance" OR "targeted surveillance" OR "hospital-acquired infection surveillance") ) AND (SU=("intensive care unit" OR "ICU ward" OR "critical care medicine" OR "ICU" OR "intensive therapy unit") OR TI=("intensive care unit" OR "ICU ward" OR "critical care medicine" OR "ICU" OR "intensive therapy unit")))

Weipu database: ( (T=("catheter-related infection" OR "catheter-associated bloodstream infection" OR "device-associated infection" OR "central venous catheter bloodstream infection" OR "CLABSI") OR K=("catheter-related infection" OR "catheter-associated bloodstream infection" OR "device-associated infection" OR "central venous catheter bloodstream infection" OR "CLABSI") ) AND (T=("infection surveillance" OR "targeted surveillance" OR "hospital-acquired infection surveillance") OR K=("infection surveillance" OR "targeted surveillance" OR "hospital-acquired infection surveillance")) AND (T=("intensive care unit" OR "ICU ward" OR "critical care medicine" OR "ICU" OR "intensive therapy unit") OR K=("intensive care unit" OR "ICU ward" OR "critical care medicine" OR "ICU" OR "intensive therapy unit") ) )
